# Physical-chemical and microbiological changes in Cerrado Soil under differing sugarcane harvest management systems

**DOI:** 10.1186/1471-2180-12-170

**Published:** 2012-08-08

**Authors:** Caio TCC Rachid, Marisa C Piccolo, Deborah Catharine A Leite, Fabiano C Balieiro, Heitor Luiz C Coutinho, Jan Dirk van Elsas, Raquel S Peixoto, Alexandre S Rosado

**Affiliations:** 1 Center for Nuclear Energy in Agriculture, Universidade de São Paulo, Av. Centenário, 303, Piracicaba, 13416-000, Brazil; 2 Embrapa Solos, Rua Jardim Botânico, 1024, Rio de Janeiro, 22460-000, Brazil; 3 Department of Microbial Ecology, University of Groningen, Groningen, 9700 CC, The Netherlands; 4 Laboratory of Molecular Microbial Ecology. Institute of Microbiology Paulo de Góes, Federal University of Rio de Janeiro (Cidade Universitária), Rio de Janeiro, 21941-590, Brazil

**Keywords:** Cerrado, Sugarcane, Soil microbiology, Nitrogen cycle, DGGE, Integrated analysis

## Abstract

**Background:**

Sugarcane cultivation plays an important role in Brazilian economy, and it is expanding fast, mainly due to the increasing demand for ethanol production. In order to understand the impact of sugarcane cultivation and management, we studied sugarcane under different management regimes (pre-harvest burn and mechanical, unburnt harvest, or *green cane*), next to a control treatment with native vegetation. The soil bacterial community structure (including an evaluation of the diversity of the ammonia oxidizing (*amoA*) and denitrifying (*nirK*) genes), greenhouse gas flow and several soil physicochemical properties were evaluated.

**Results:**

Our results indicate that sugarcane cultivation in this region resulted in changes in several soil properties. Moreover, such changes are reflected in the soil microbiota. No significant influence of soil management on greenhouse gas fluxes was found. However, we did find a relationship between the biological changes and the dynamics of soil nutrients. In particular, the burnt cane and green cane treatments had distinct modifications. There were significant differences in the structure of the total bacterial, the ammonia oxidizing and the denitrifying bacterial communities, being that these groups responded differently to the changes in the soil. A combination of physical and chemical factors was correlated to the changes in the structures of the total bacterial communities of the soil. The changes in the structures of the functional groups follow a different pattern than the physicochemical variables. The latter might indicate a strong influence of interactions among different bacterial groups in the N cycle, emphasizing the importance of biological factors in the structuring of these communities.

**Conclusion:**

Sugarcane land use significantly impacted the structure of total selected soil bacterial communities and ammonia oxidizing and denitrifier gene diversities in a Cerrado field site in Central Brazil. A high impact of land use was observed in soil under the common burnt cane management. The green cane soil also presented different profiles compared to the control soil, but to at a lesser degree.

## Background

Sugarcane is an efficient substrate for bioethanol production, wich is currently largely used in Brazil as a substitute for fossil fuels. Traditionally, sugarcane crops are burnt before harvest, in order to remove leaves, thus facilitating easier manual harvest. However, this procedure results in high emissions of particulate matter and smoke, which can be harmful to humans and livestock. Current regulation of bioethanol production is leading to a transition towards mechanical harvest. Several authors have reported the positive effects of unburnt harvest (green cane) on soil fertility, soil structure, soil C levels and biological activity [1-3]. Most of these data have been generated in studies in the Atlantic Forest biome, however none has addressed the microbial community structures and diversities in soils under burnt versus green cane management in Cerrado Biome.

The Cerrado is the second largest terrestrial biome in Brazil and it is characterized by a savannah-like vegetation on ancient and plain soils [4]. Currently, cultivation of sugarcane is increasing in this region, with some states showing a 300% expansion of cropped areas over the last few years [5]. Due to high concentrations of endemic plant species and the accelerated pace of deforestation, the Cerrado region has been classified as a high priority area for biodiversity conservation [6]. Therefore, there is a need to develop studies that address the effects of sugarcane expansion in Cerrado soils.

The use of agricultural land for cropping generally results in modifications of the soil biological and physicochemical properties, which, in turn, affect soil biogeochemical processes such as nutrient cycling and gas emissions, influencing ecosystem productivity and sustainability [7-11]. Brazil is the fifth largest contributor to the global emission of greenhouse gases (GHG). A major part, up to 75%, is the consequence of unsustainable agricultural practices next to deforestation, which include removal of crop residues, exposure of the soil surface to erosion, excessive plowing and the introduction of nitrogen fertilizers in excess [12-14]. In areas under sugarcane, there is no consensus regarding the effects of burning or maintenance of crop residues on the soil microbiota and the emission of GHG, nor on the chemical and physical factors of the soil [15-17].

Some soil properties respond relatively rapidly to land use and soil management changes, which makes these suitable to serve as soil quality indicators [18]. For instance, the light, labile fraction of soil organic matter, dissolved C and N contents, soil microbial biomass and activity, and bacterial diversity, have all been proposed to represent suitable early warning indicators of soil quality degradation or improvement [2,11,19-23]. However, we are far from having a consolidated set of soil quality indicators, which might allow such monitoring across a range of different soils [24,25].

Specific groups, such as ammonia oxidizing and denitrifying bacteria, play basic roles in the N cycling. The study of these groups is very important, mainly in agricultural soil, since nitrification coupled with denitrification are major sources of soil N loss. The use of molecular tools targeting key genes such as *amoA* and *nirK* have been widely used to improve the knowledge about this issue. Their ecology can be more readily understood by exploring the abundance and diversity of key marker genes than through cultivation based approaches [26].

The great majority of studies on effects of different cropping systems evaluates just one or a few parameters in soil; thus, stable isotopes are used to better understand C and N dynamics [3], bacterial communities to establish soil quality bioindicators [17] and greenhouse gas fluxes to evaluate impacts on global warming [15]. On top of this, there is a paucity of knowledge with regard to parameters that might serve as quality indicators for Cerrado soil under sugarcane cultivation, that is, what parameters might serve as quality indicators.

Since physical, chemical and biological factors in soil are not independent from each other, it is important to evaluate them together in one system and to attempt to establish the links between them. The main goal of our study was therefore to evaluate the impact of the different management strategies of sugarcane (burnt cane and green cane) on the soil chemical, biological and physical properties (including GHG flow) and to analyze the relationships between these features.

## Methods

### Field site

The study area (17° 55' 35" S 50° 08' 36" W) was located in the municipality of Porteirão, state of Goiás, Brazil. The region´s climate is classified as Aw (Köppen), with annual average rainfalls exceeding 1500 mm year^-1^ and annual average air temperatures of 23.1°C. The soil type is a eutrophic *Latossolo vermelho* (Ferralsols), which is characterized by high levels of base saturation (>50%). Although the area was very flat, petroplinthite (lateritic nodules or concretions) were found in the subsurface, which may restrict drainage and exhibits a concretionary character.

The field had been previously used for cotton, soy and sunflower production, and was converted to sugarcane cultivation in 2002. The samples were collected in September 2008, during the sugarcane growth stage, approximately 7 to 8 months after bud germination (after six yearly harvest cycles). The field was divided into three treatments (split-plot) in which three different regimes were applied:

(i) Burnt sugarcane – Before harvest, the sugarcane crop was burnt to remove the leaves. The stem was then manually harvested. After harvest, the soil remained uncovered.

(ii) Green sugarcane – Harvest was performed using a machine that separates the sugarcane leaves from the stems. The leaves are then returned to the soil. After harvest, the soil remained covered by the vegetal residues.

(iii) Control – covered with trees interspersed with open areas, contiguous to the sugarcane treatments.

The sugarcane treatments had 6 years of implementation until the sampling. The fertilization regime of the area was composed by the addition of 400 kg ha^-1^ of NPK (5-25-15) during the implementation of the sugarcane crop (6 years before the sampling), and an annual addition of 400Kg of NPK (20-0-20), after each harvest (8 months before the sampling). Monoammonium phosphate was used as nitrogen source during the first fertilization and urea in all other subsequent ones. To allow replication, per treatment, five 5x5m subplots were defined randomly (approximately 10 m of distance from each other). The soil was collected as five replicates per subplot (which were pooled) approximately to 10 cm depth, using a core borer (total up to 2.5 kg). The sizes of the burnt sugarcane, green sugarcane and control treatments were 23.5, 9.9 and 2.9 ha, respectively. The native vegetation was chosen as control because it represents the soil's natural condition; it received no addition of fertilizers. This control was a small fragment of native Cerrado (Cerradão-type, characterized by a dense formation of trees up to 4 meters tall) [4]. The three treatments were very close to one another, less than 300 m apart.

### Soil physical and chemical properties

Subsamples of soils from each site were air dried, sieved (2 mm) and analyzed chemically. Exchangeable nutrients: Ca^2+^, Mg^2+^ and Al^3+^ extracted by 1 M KCl; P, Na and K by Mehlich-1 extractant – 0.05 mol L-1 in HCl in 0,0125 mol L-1 H2SO4) and pH (soil:water, 1:10); Potential acidity: H + Al extracted with calcium acetate 1 N (pH 7), titrated with 0.0125 N NaOH, were analysed according to Embrapa [27]. Inductively coupled plasma apparatus for Ca^2+^, Mg^2+^ and Al^3+^, flame emission (K and Na) and photocolometry (for P) were used for nutrient determinations. All analyses, except bulk soil density and potential denitrification (where samples were pooled), were conducted with all five replicate samples per treatment.

Soil granulometry was determined using the aerometer method, after chemical dispersion [27]. Soil bulk density (2.5-7.5 cm) was determined in undisturbed samples, collected with 5 cm diameter and 5 cm height stainless steel rings, from three samples per treatment. The water-filled pore space percentage (%WFPS) was determined by converting soil gravimetrical water content values (θ_g_ g g^-1^), using the equation:%WFPS = (100θgd)/[1 – (d/pd)], where d is the soil bulk density and pd is the particle density [28].

### Total and isotopic organic C and N contents in the soil

The isotopic organic C to N ratio was used to infer the C and N turnover in this environment. Since the previous vegetation at the sites were plants with C3 metabolism and sugarcane is a plant with C4 metabolism, we could measure the turnover of organic matter by measuring the differences in the isotopic ratio values. Soil total C and N contents and ^13^ C^/12^ C and ^15^ N^/14^ N isotopic ratio variations were determined by use of an elemental analyzer coupled to a mass spectrometer (Carlo Erba/Delta Plus). Results were expressed in the form of δ ^13^ C (‰) in relation to the international PDB standard and as δ ^15^ N (‰) in relation to the atmospheric N [29].

### Inorganic N content

On the day of sampling, inorganic N was extracted from the soil samples using a KCl (2 M) solution (time 0). Moreover, the soil was extracted after a 7-d incubation period [30]. Phenyl mercury acetate (0.1 mL) was added to the filtrate to preserve the samples. The ammonium (NH_4_^+^) and nitrate (NO_3_^-^) contents in the extracts were determined using an automatic flow injection analysis system. Ammonium was quantified colorimetrically using the Solorzano method [31], and nitrate estimated by conductivimetry in the form of nitrite (NO_2_^-^), after reduction with a cadmium base catalyst [32]. The net N mineralization rates of the soil samples were calculated by the difference between the concentrations of NH_4_^+^-N and NO_3_^-^-N before and after 7 days of incubation. The net nitrification rates were calculated by the differences between final and initial NO_3_^-^-N contents in the incubated soil samples.

### Gas fluxes

To determine the fluxes of CO_2_, N_2_O and CH_4_, gaseous samples were collected from 10-L static chambers installed in the field. We installed six chambers per treatment, and samplings were done for three consecutive days (at 10 p.m.). Thus, in the sugarcane treatments, to cover the different soil conditions in relation to the plant influence on gas flux, two chambers were placed along the cultivation rows, two in between the rows (0.45 m from the row) and two in an intermediate region between the rows and the space between the rows (0.225 m from the row). The samples were obtained through nylon syringe (50 mL; BD) at intervals of predetermined time (1, 10, 20 and 30 minutes). The gas collected was immediately transferred to glass vials (20 ml) pre-evacuated and sealed for storage and further analysis. The N_2_O concentration was determined with an electron capture detector (ECD) detector, using a Haysep Q 3 m, 1/8” column and the CO_2_ and CH_4_ concentrations were determined with a flame ionization detector (FID) detector using a Porapak Q 2 m, 1/8” column. The gas fluxes were calculated by the change in the concentration of the gases inside the chambers over the incubation period (30 min).

### Soil potential denitrification rates

Denitrification rates were determined as described by Smith and Tiedje [33]. Fifty grams of soil were incubated in hermetically sealed glass (1.8 L) bottles, containing a nutrient solution with NO_3_^-^ (100 mg N l^-1^), glucose (40 mg l^-1^) and chloramphenicol (10 mg l^-1^). The atmosphere in the bottle was replaced by pure N_2_ and approximately 10% of acetylene was added. Gas samples were removed after 0, 30, 60 and 90 min. Tests were conducted in triplicate. The N_2_O concentrations were quantified with a gas chromatograph (Shimadzu GC17A).

### Bacterial community structure and N cycle gene diversity

Soil DNA was extracted in triplicate (only three soil samples randomly chosen from the five replicate subplots) by using the FastDNA® Spin Kit for Soil and a FastPrep® equipment (Bio 101, CA, USA), according to the manufacturer’s instructions.

To analyze total bacterial community structure and diversity, we used a pair of universal primers for the domain Bacteria, which amplify the gene fragment coding for a fragment of the 16 S rRNA subunit (U968-GC and L1401) [34]. Specific primers for the functional genes *amoA* (AmoA1F-Clamp and AmoA-2R-TC) [35] and *nirK* (F1aCu and R3CuGC) [26] were used to study the ammonia oxidizing and denitrifying bacteria, respectively. A CG-rich clamp was added to the end of one primer for each system [36].

Amplifications were carried out by PCR in 50 μL reactions containing approximately 10 ng of DNA, *Taq* buffer 10X, MgCl_2_ (2.5 mM), dNTPs (0.2 mM), primers (0.2 μM), BSA (bovine serum albumin) (0.1 g l^-1^), formamide (1% v/v) and *Taq* DNA polymerase (Fermentas; 2.5 U).

The bacterial PCR was run as follows: initial DNA denaturation step at 94°C for 4 min, followed by 35 cycles of 1 min at 94°C, an annealing step of 1 min at 55°C, and amplification during 2 min at 72°C, with a final extension of 10 min at 72°C. The *amoA* gene-specific PCR was run with an initial denaturation at 94°C for 3 min, followed by 35 cycles of 30 s at 94°C, 1 min at 57°C, 1 min at 72°C, with a final extension of 10 min at 72°C. The denitrifying gene-specific PCR was run with an initial denaturation at 94°C for 3 min, followed by 5 cycles of 30 s at 94°C, 1 min at 60°C and 1 min at 72°C; 30 cycles of 30 s at 94°C, 1 min at 62°C, and 1 min at 72°C; with a final extension of 10 min at 72°C.

The amplified fragments were analyzed via DGGE [37] on a Universal Dcode™ Mutation Detection System (Bio-Rad, Richmond, California, USA). We prepared the polyacrylamide gels (6%) using a mixture of 37.5:1 acrylamide/bisacrylamide (w:w) in a TAE 1X buffer (10 mM Tris-acetate, 0.5 mM EDTA pH 8.0), with denaturing gradients of: 45 to 65%, 45 to 65%, and 55 to 70%, for bacterial, ammonia oxidizing and denitrifying gene amplicons, respectively.

Approximately 30 μL of the PCR products (approximately 20–30 μg of DNA) were applied to each slot, and electrophoresis was performed at 75 V at 60°C for 16 h. Gels were stained with SybrGreen® (Molecular Probes, Oregon, USA) and observed on a Storm® scanner (GE Healthcare).

### Data analysis

All data were tested for normality and homoscedasticity. When these conditions were met, analysis of variance (ANOVA) followed by Tukey tests for the significance of the differences were used. Otherwise, the non-parametric Kruskal-Wallis ANOVA & Median, followed by two-sided Kolmogorov-Smirnov tests were applied. All analyses were performed using the program STATISTICA 7 (StatSoft).

To analyze the difference between microbial community structures, N transformation gene diversities, and their interactions with abiotic factors, we used non-metric scaling (NMS) with the aid of the PC-ORD statistical package V5 (MjM Software, Gleneden Beach, OR).

Matrices containing all physicochemical properties and bacterial community and functional gene data were assembled to carry out the ordinations. The DGGE band profiles were digitalized and inserted into the data matrices by use of the Bionumerics v6.0 package (Applied Maths), according to the manufacturer’s instructions.

The matrices were ordered by NMS [38,39], employing a Bray-Curtis distance matrix. NMS was performed using a random initial configuration, and the data matrices were analyzed using 250 runs with real data and compared with the Monte Carlo test with 250 runs of random data. The final result of the NMS analyses was restricted to two dimensions to simplify data analyses and discussions (stability criterion = 0.00001; interactions to evaluate stability = 15; maximum number of interactions = 250). The stability of the standards of ordination in reduced size was developed by plotting the values of stress by numbers of interactions. Despite the fact that all variables are present in the ordination analysis, only those that were significantly correlated with the microbial ordination are presented.

To confirm the existence of the groupings generated by NMS analysis we performed a Multi-Response Permutation Procedure (MRPP) that tests the hypothesis that no difference exists between two or more groups of entities [40].

To evaluate the association between the generated matrix and the data from the physicochemical properties and the matrices from the DGGE profiles, we used a Mantel test [41], which evaluates the hypothesis that a relationship between two matrix distances does not exist. All Mantel tests were employed using the asymptotic approximation of Mantel and the Sørensen distance [42].

## Results and discussion

### Soil chemical and physical properties

The three field sites studied were homogeneous and belonged to the same soil class. Briefly, all three sites were very similar in their mineralogical composition, constituted mainly by kaolinite, gibbsite, hematite and goethite (data not shown). The clay content was variable across the samples of all three fields, between 300 (minimum) and 480 g Kg^-1^ (maximally). Other soil properties differed between the fields where the different treatments were applied (Table 1). Thus, the burnt soils showed slight acidification and decrease of exchangeable Ca, exchangeable Mg, total P and SB (sums of bases) values and CEC (cation exchange capacity) levels.

**Table 1:** Average values of soil properties

**Parameter**	**Treatment**		
	**Control**	**Green cane**	**Burnt cane**
pH	6.6^a^	6.4^a^	5.9^b^
Exchangeable Al	BD	BD	BD
Exchangeable Ca	11.4^a^	10.^b^	4.3^c^
Exchangeable Mg	3.9^a^	2.1^b^	1.6^c^
Exchangeable Na	1.7^a^	2.8^a^	BD
Exchangeable K	306.6^b^	735.6^a^	280.0^b^
Exchangeable H + Al	4.8^b^	5.0^b^	6.5^a^
Total P	102.3^a^	34.6^ab^	32.6^b^
SB^1^	16.1^a^	14.2^b^	6.6^c^
CEC^2^	20.9^a^	19.0^b^	13.1^c^
V^3^	77.0^a^	74.7^a^	50.4^b^
Bulk density	0.96 ^b^	1.25^a^	1.31^a^
Moisture	29.2^a^	26.2^a^	27.6^a^
WFPS^4^	41.8 ^b^	58.7^a^	64.9^a^
Total C	12.5^a^	6.7^b^	15.9^a^
Total N	0.70^a^	0.30^b^	0.90^a^
δ^13^C	−22.8^a^	−20.9^b^	−23.1^a^
δ^15^N	8.8^b^	11.4^a^	8.3^b^
C:N	17.9^b^	22.3^a^	16.4^b^

The numbers represent average values (n = 3 for density and n = 5 for the rest). Averages followed by the same letter in each line are not statistically different (5%) from each other according to the Kolmogorov-Smirnov test for Ca, Mg, Na, K, P and V; and according to the Tukey test for the rest. BD - Below the detection limit of the technique. ^1^Sum of bases (sums of the Ca, Mg, Na and K content in cmol_c_ dm^-3^). ^2^Cation exchange capacity (sums of SB and H + Al). ^3^Percent base saturation (SB divided by CEC). Parameters units: Al, Ca, Mg, H + Al, P, SB, CEC (cmol_c_ dm^-3^), Na, K (mg dm^-3^), V (%), Bulk density (g kg^-1^), δ^13^C, δ^15^N (‰). ^4^ Water filled pore space.

Moreover, significant differences between treatments regarding soil bulk density and water filled pore space (WFPS) were noted. Both green and burnt cane soils had significantly higher bulk densities as compared to the control, i.e. 1.25 and 1.31, respectively, versus 0.96. We did not observe any major differences in soil moisture content, although the control showed a significantly decreased WFPS value (Table 1).

The increase of soil bulk density under sugarcane cultivation is commonly observed when soil passes from its natural to a cultivated condition [3]. It occurs due to the breaking up of aggregates caused by soil tilling, the use of agricultural machines and the loss of organic matter [43].

### Soil C and N content

The data showed lower values for total C and total N in the green cane (p < 0.05) versus the burnt treatment. In addition, the C:N ratio was significantly higher in the green cane soil (Table 1) than in other treatments. Moreover, raised values of δ^13^C and δ^15^N were observed in green cane, in comparison with the other treatments. Collectively, these data suggested that, in the green cane soil, a larger contribution to soil organic matter was provided by sugarcane (C4 photosynthetic cycle plant), next to a more intense and open N cycling.

The lower C and N contents in the green cane soil were unexpected, and appear to contradict previous reports [3]. However, other studies on different cultivation and agricultural management systems [44], including sugarcane [45], reported that the recovery of the soil C stock depends on the time elapsed after changes in the agricultural practices were made. The relatively short time given in the current study to the green cane management was likely insufficient to positively affect the C content in the soil. Possibly, during the transition to this system, more labile organic matter was incorporated than that incorporated in the form of burnt compounds, resulting in higher soil respiration rates, which may have reduced C contents in this treatment. Moreover, the maintenance of crop residues may have created better conditions for microbial activity, resulting in an increased cycling of soil organic matter. This hypothesis is supported by the higher values of δ^13^C and δ^15^N found in the respective soil (Table 1). The soil δ^13^C detected in all treatments was between −20‰ and −23‰, suggesting that the soil OM is a combination of the OM from previous cultivation (C3 plants) and also from the current sugarcane cultivation (C4 plants). However, the more enriched signal found in green cane indicates that the detected C derives primarily from the C4 route. Moreover, the higher δ^15^N also indicates a more intense N cycling.

The C contents of the soil under the two regimes were on the order of those found in other sugarcane plantings [3]. However, studies in the same soil under natural vegetation or agricultural use previously reported higher organic C contents [46,47]. Further studies should attempt to assess the extent to which land use affects soil C stocks.

Ammonium was the predominant form of mineral N in the control soil, whereas the two soils under sugarcane showed a predominance of nitrate (Table 2). Such changes of the predominant soil N form promoted by land use change have been reported earlier [10]. With respect to the N cycle, the net rates of N mineralization and nitrification were significantly lower in the two soils under sugarcane cultivation, when compared with the control (Table 2). Such effects of the use of soil have been observed before [10,48,49]. However, the changes in sugarcane harvest management did not result in an alteration of the patterns of N transformations, agreeing with previous published results [50].

**Table 2:** **Contents of NH**_**4**_^**+**^-N**, NO**_**3**_^**-**^-N**, net rates of N mineralization and nitrification in the soil and denitrifier enzyme activity (DEA) of the soil (0–10 cm)**

**Treatment**	**NH**_**4**_^**+**^**-N**	**NO**_**3**_^**-**^**-N**	**Mineralization**	**Nitrification**	**DEA**
	**mg kg**^**-1**^**dried soil**		**mg kg**^**-1**^**dried soil day**^**-1**^		
Control	9.6 (1.5)^a^	1.3 (0.5)^b^	2.6 (0.5)^a^	2.6 (0.4)^a^	2.6 (0.3)^a^
Green cane	13.5 (12.1)^ab^	32.6 (27.9)^a^	−4.2 (6.0)^b^	−2.5 (3.9)^b^	0.1 (0.0)^b^
Burnt cane	1.9 (0.9) ^b^	26.6 (15.9)^a^	−0.5 (0.8)^b^	0.4 (0.8)^b^	0.1 (0.0)^b^

The numbers represent average values (n = 3 for DEA and n = 5 for the rest) followed by their respective standard deviations in parentheses. Averages followed by the same letter in each column are not statistically different from each other according to the Tukey test (5%) for DEA and the Kolmogorov-Smirnov test (5%) for the rest.

Variations in the NH_4_^+^-N:NO_3_^-^-N ratio values may result from distinct processes [51]. In our study, the main factor that interfered with the ratio values was the denitrification rate. As the highest rate of nitrification, found in the control soil, was associated with higher ammonium content, this is not the most plausible mechanism. Additionally, the potential soil denitrification rates were higher in the control soil, as compared to the two planted treatments (Table 2). The suppression of the soil potential denitrificaton rate can provide higher N-NO_2_ content, and could be explained by a shift in soil microbiology. Denitrification enzyme activity (DEA) value distributions correlated significantly (p < 0.01) with changes in the soil bacterial community and ammonia oxidizing and denitrifiers gene structures. It corroborates work of other authors that stressed the link between shifts on specific bacterial communities with changes in the denitrification process [52,53].

### Greenhouse gas fluxes

**Figure 1:**
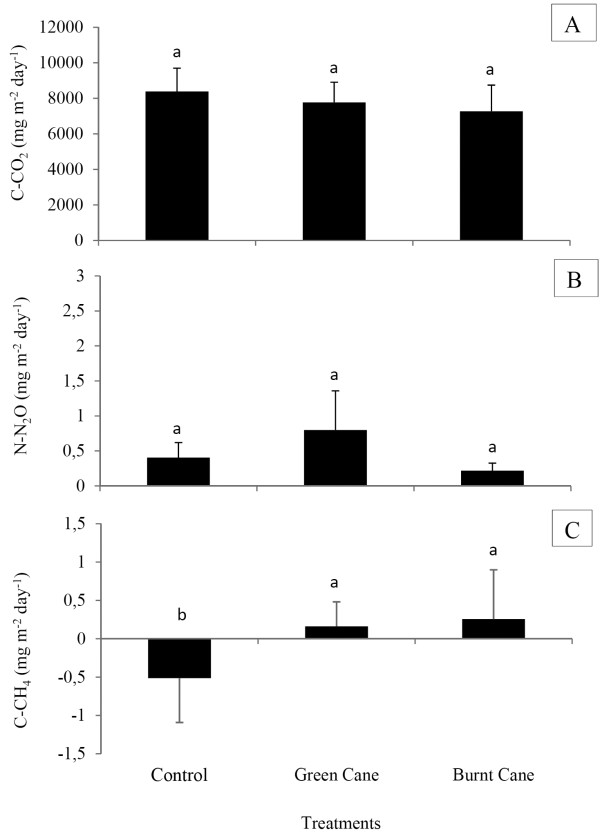
**Flux of C-CO**_**2**_**(a), N-N**_**2**_**O (b) and C-CH**_**4**_**(c) proceeding from soils.** The graphics represents the average flux (n=18) and the bar represents its standard deviation. The same letters indicate values that are not statistically different from each other according to the Tukey test (5%) for CO_2_ and the Kolmogorov-Smirnov test (5%) for CH_4_ and N_2_O.

We analyzed the in situ fluxes of several selected gases to understand the effect of land use on greenhouse gas production. The data showed that the N_2_O and CO_2_ fluxes had similar behavior (Figure 1), and differences were not observed between the different treatments. However, the flux of methane suffered an inversion in its direction in both sugarcane soils (Figure 1).

Probably, the lower density and WFPS measured on Cerrado plays an important role in the flux dynamics for CH_4_ and N_2_O gas, because it means that the Cerrado Soil (letra maiuscula) offers a more aerobic environment, inhibiting both methane production and denitrification enzyme activity. However, the fluxes of N_2_O and methane were low in the period of measurement, and therefore might be negligible as contributors to greenhouse gas emission, even considering their higher effect on global warming.

Regarding the spatial variation of the fluxes within the sugarcane cultivated soils, higher emissions were detected in the chambers that had been placed on the planted rows when compared with the region between the rows (data not shown), showing the influence of the rhizospheric soil and the root respiration. It is important here to point out that these conclusions were obtained from a single sampling of three days. To confirm the observations, a more comprehensive study including different sampling times, possibly over different seasons, is needed.

### Structures of the bacterial communities and their relationship with soil properties

**Figure 2:**
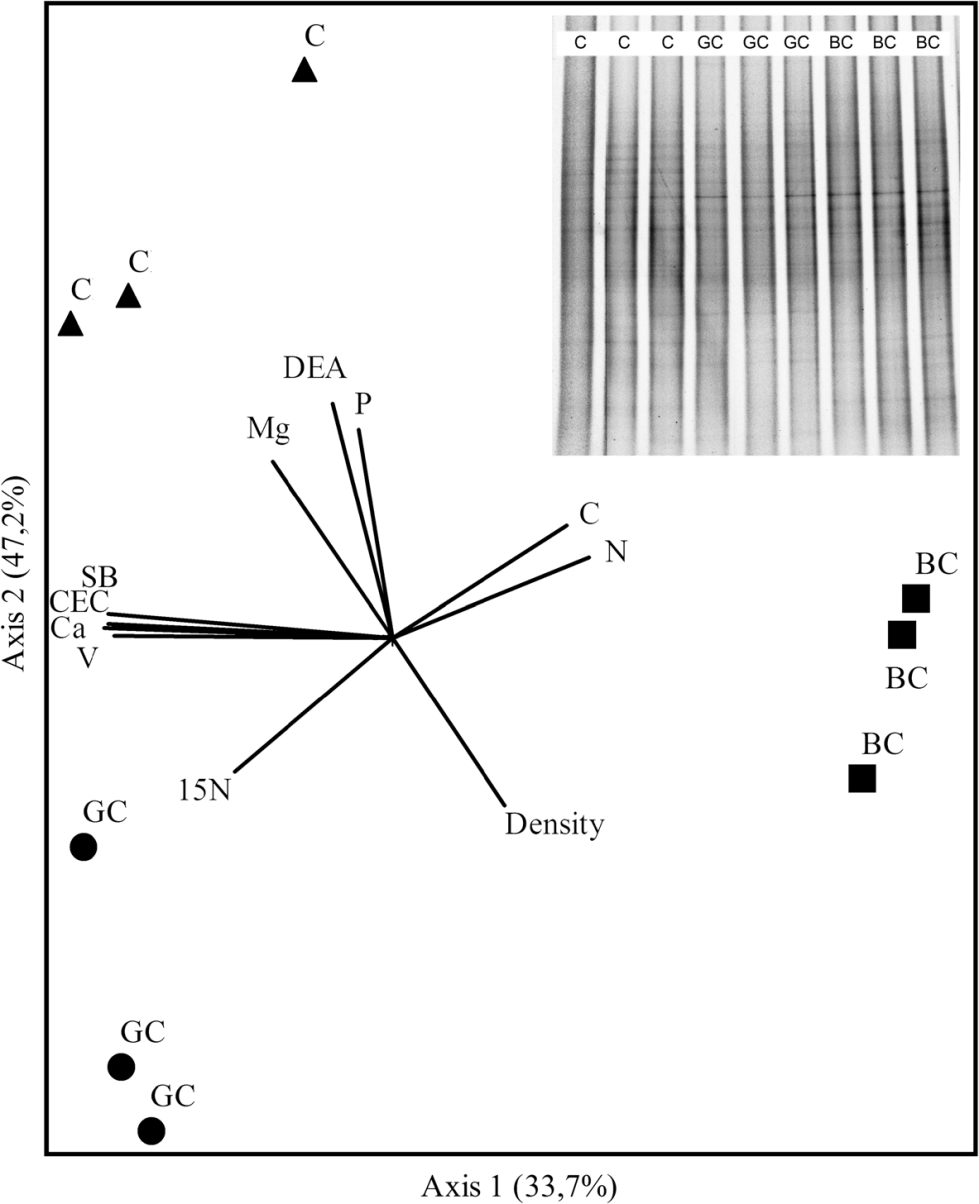
**NMS ordination of the DGGE profiles of 16S rRNA gene fragments (total bacteria) amplified from the soil samples (0–10 cm) collected from the treatments Control (C), Green cane (GC) and Burnt cane (BC).** The fraction of total variance that accounts for each axis is indicated in parentheses. The angles and the length of radiating lines indicate the direction and strength of the relationship between the chemical and biological variables with the ordination scores.

The bacterial DGGE profiles of all treatments were complex, with high numbers of bands and no clear dominance. The profiles of the three samples of each treatment revealed great similarity. The analyses of the structure of the bacterial communities (Figure 2) showed that these were significantly impacted by both the use (cultivation of sugarcane) and the management (burnt versus green cane) of the soil, according to pairwise comparisons (MRPP analysis; p < 0.03). The ordering generated by the NMS grouped the replicates of each treatment in a distinct region, and the three treatments (centroids) practically equidistant from each other. The sensitivity of soil bacterial communities to changes in land use and management has already been shown by different authors in various settings [11,54-56], including DGGE analyses carried out in Brazilian Cerrado soils [20].

Several factors correlated with the NMS ordination. In particular, the total P and exchangeable Mg contents and soil density were associated with the bacterial community structures in the control soil, while the (reduced) C and N contents were correlated with the bacterial communities in the green cane treatment. Finally, the (decreased) value of the sum of bases (SB), the degree of saturation of the bases (V), the cation exchange capacity (CEC) and exchangeable calcium (Ca) were correlated with the communities from the burnt cane treatment (Figure 2). The soil properties that correlated with the segregation of the bacterial community structures were consistent with observations from Atlantic forest soils under different agricultural production systems [11,17,20].

**Figure 3:**
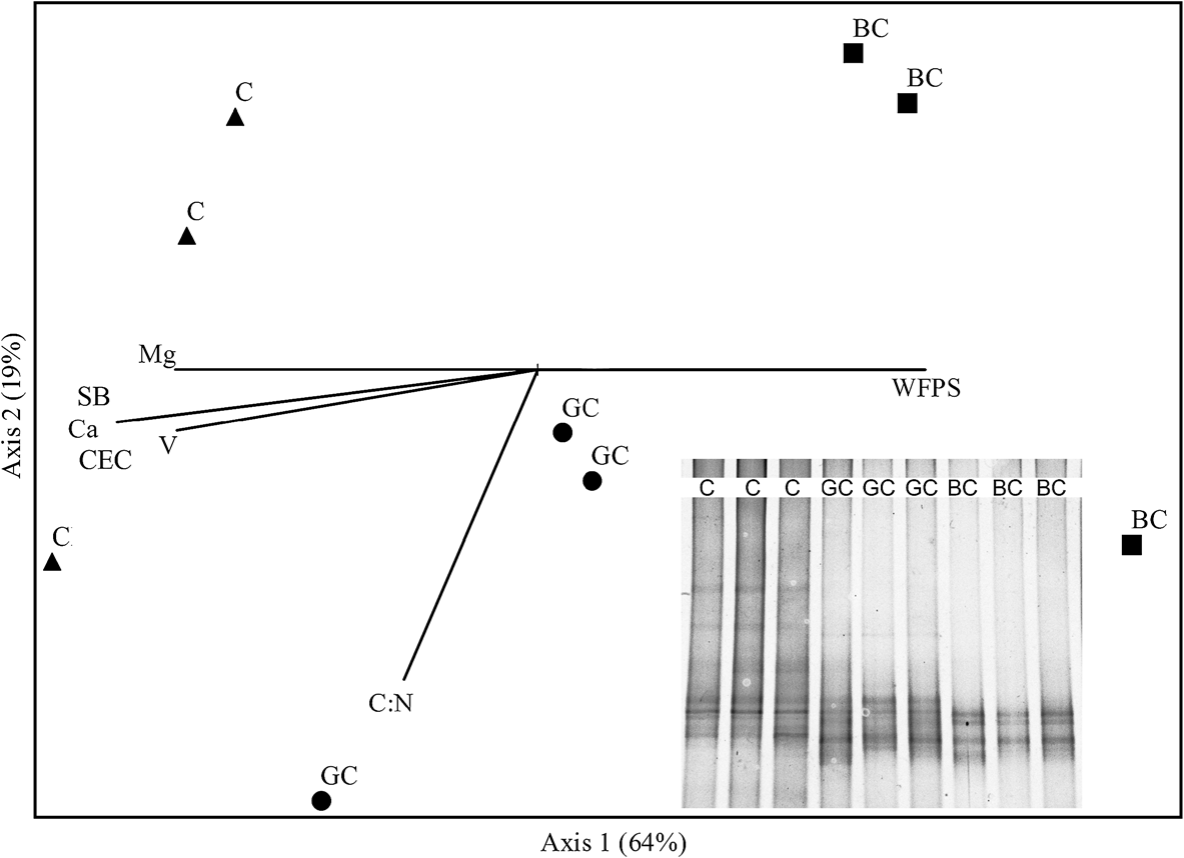
**NMS ordination of the DGGE profiles of *amoA* gene fragments (ammonia oxidizing bacteria) amplified from the soil samples (0–10 cm) collected from the treatments Control (C), Green cane (GC) and Burnt cane (BC).** The fraction of total variance that accounts for each axis is indicated in parentheses. The angles and the length of radiating lines indicate the direction and strength of the relationship between the chemical and biological variables with the ordination scores.

The *amoA* gene based DGGE (ammonia oxidizing bacteria) showed relatively simple profiles in all treatments (4–10 bands), with relatively similar patterns between the triplicates. The control soil revealed a higher number of bands in comparison to the green and burnt cane soils. The analysis of these communities indicated a diffuse distribution, with some within-treatment variability (Figure 3). However, as reflected in the X axis, these communities responded significantly to the change in land use management (MRPP < 0.05), being the burn treatment a factor that exacerbated the response.

None of the parameters tested correlated with the grouping of the *amoA* communities in the green cane soil, with the exception of the C:N ratio in one replicate. The clear distinction between the bacterial communities in the control soil and in the burnt cane soil was correlated with the high exchangeable Mg content and the low WFPS value in the former. Moreover, it was associated with low values of the sum of bases, cation exchange capacity, exchangeable Ca and the degree of saturation of the bases in the burnt cane soil (Figure 3).

**Figure 4:**
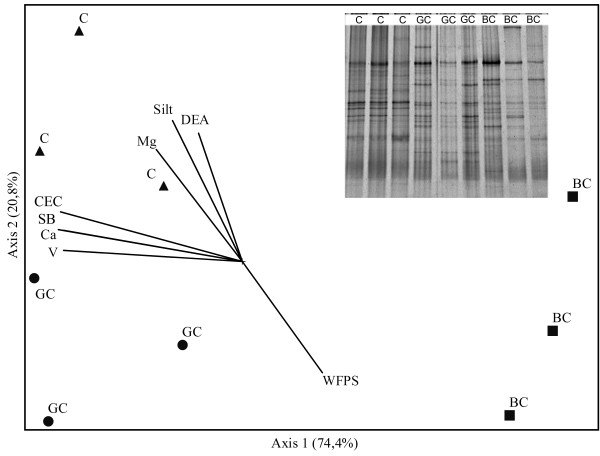
**NMS ordination of the DGGE profiles of *nirK* gene fragments (denitrifier bacteria) amplified from the soil samples (0–10 cm) collected from the treatments Control (C), Green cane (GC) and Burnt cane (BC).** The fraction of total variance that accounts for each axis is indicated in parentheses. The angles and the length of radiating lines indicate the direction and strength of the relationship between the chemical and biological variables with the ordination scores.

The *nirK* gene based DGGE profile (denitrifying bacteria) showed more complex patterns (8–15 bands) than that of the ammonia oxidizing bacteria. The triplicate profiles were similar between each other. Much like the total bacteria, the *nirK* based patterns (Figure 4) showed significant differences between treatments (MRPP < 0.03). However, there was great variation in community structure. There was a distinction between green cane and control samples along the Y axis and a marked distinction between the burnt cane and the other samples along the X axis, that contained the major percentage of variance (74%).

None of the soil parameters tested showed significant correlation with the alterations in the structure of the denitrifying community in the green cane soil. In the burnt cane soil, the factors involved in the process were the same as described above. The communities in the control soil were also strongly influenced by the high exchangeable Mg value and the low WFPS (Figure 4).

Ordination of the physicochemical data as primary matrices classified the treatments as three distinct groups (data not shown), which is the same basic grouping found with the bacterial community. In contrast, the two functional communities did not follow the same pattern as the bacterial communities, perhaps because these groups were subjected to more specific selective forces, such as caused by different levels of NH_4_^+^-N and/or NO_3_^-^-N. The Mantel correlation data (not shown), that test the correlation and the significance between two matrices, provided evidence for the latter hypothesis, because the largest correlation value found was that of the ammonia oxidizing community with the denitrifier community (r = 0.70), while the correlation of these groups with soil properties was respectively at r = 0.45 and r = 0.63. In spite of the fact that all correlations were significant (p < 0.01), the high value found between the two groups of N cycle bacteria emphasized the interdependence of the two different bacterial groups involved in the N cycle with soil N chemistry. It may hint at the importance of biological factors in the structure of these communities. A change in density, reflected in the respective community, may directly affect the others. Du et al. [57] also demonstrated (*in vitro*) a strong correlation between ammonia oxidizing and denitrifier bacteria, and this relationship can apparently also be detected in agricultural soil.

## Conclusion

Sugarcane land use significantly impacted the structure of soil bacterial communities and ammonia oxidizing and denitrifier gene diversity in a Cerrado field site in Central Brazil, with significantly correlations (p ≤ 0.01) with several soil properties. Different factors, but especially the DGGE and the DEA activities were very sensitive to the management practices. A high impact of land use was observed in soil under the common burnt cane management, where the shifts were correlated with soil bulk density and water-filled pore spaces. The green cane soil had also changed from the control soil, but to at a lesser degree. Both treatments showed positive correlations between the make-up of the respective communities and soil fertility indicators (sum of bases, CEC and degree of base saturation), with the green cane treatment showing a negative correlation with C and N contents in the bacterial community structure, possibly due to increased biological activity and C oxidation.

Given the fact that soil nitrification is known to be a phylogenetically restricted process, it is important to assess the effects of land use on its diversity. We here found that the use of Cerrado soil for sugarcane cropping results in a community structure shift as compared to a control treatment. Importantly, the burn treatment resulted in the largest change in this microbial structure for both ammonia oxidizing and denitrifying gene diversity, as could be noted by the reduction of band numbers in the DGGE profiles and higher community differentiation on NMS analysis. We believe that answers obtained by the evaluation of bacterial community structure can be as important as the number of microorganisms, and that is important to quantify the size of these communities in this environment. Therefore, a complex study to answer this question is being carried on.

It is clear that we have provided just a snapshot of potential changes in soil resulting from the changed management (burnt to green cane). Thus, further research is required in which soil samples from different sites of the Cerrado are used, possibly comprising different seasons, in order to address the changes due to changes in management over the years.

## Competing interests

The authors declare that they have no competing interests.

## Authors’ contributions

CTCCR: Conception and design, field work, laboratory analysis, data interpretation and analysis, manuscript writing; MCP: Conception and design, field work, coordination, data interpretation and manuscript review; DCAL: Laboratory analysis; FCB: Conception and design, field work, data interpretation and manuscript writing; HLCC: Data interpretation and manuscript review; JDVE: Data interpretation and manuscript writing; RSP: Data interpretation and manuscript writing; ASR: Data interpretation, coordination and manuscript review. All authors read an approved the final draft.
